# Association of Glucose Metabolism and Blood Pressure during Pregnancy with Subsequent Maternal Blood Pressure

**DOI:** 10.1038/s41371-020-00468-2

**Published:** 2021-02-03

**Authors:** M. Maresh, J.M. Lawrence, D.M. Scholtens, A. Kuang, L.P. Lowe, C. Deerochanawong, D.A. Sacks, W.L. Lowe, A.R. Dyer, B.E. Metzger

**Affiliations:** 1 Manchester University NHS Foundation Trust, Manchester Academic Health Science Centre, Manchester, United Kingdom; 2 Kaiser Permanente Southern California, Pasadena, CA, USA; 3 Northwestern University Feinberg School of Medicine, Chicago, IL, USA; 4 Rajavithi Hospital, Bangkok, Thailand

**Keywords:** pregnancy, gestational diabetes, hypertension, insulin resistance, BMI

## Abstract

The goal of this study was to examine associations of measures of maternal glucose metabolism and blood pressure during pregnancy with blood pressure at follow up in the Hyperglycemia and Adverse Pregnancy Outcome (HAPO) cohort. The HAPO Follow-Up Study included 4747 women who had a 75-g oral glucose tolerance test (OGTT) at ~28 weeks’ gestation. Of these, 4572 women who did not have chronic hypertension during their pregnancy or other excluding factors, had blood pressure evaluation 10–14 years after the birth of their HAPO child. Primary outcomes were systolic blood pressure (SBP), diastolic blood pressure (DBP) and hypertension (SBP ≥140 and/or DBP≥90 or treatment for hypertension) at follow-up.

Blood pressure during pregnancy was associated with all blood pressure outcomes at follow-up independent of glucose and insulin sensitivity during pregnancy. The sum of glucose z-scores was associated with blood pressure outcomes at follow-up but associations were attenuated in models that included pregnancy blood pressure measures. Associations with SBP were significant in adjusted models, while associations with DBP and hypertension were not. Insulin sensitivity during pregnancy was associated with all blood pressure outcomes at follow-up, and although attenuated after adjustments, remained statistically significant (hypertension OR 0.79, 95%CI 0.68– 0.92; SBP beta −0.91, 95% CI −1.34– −0.49; DBP beta −0.50, 95% CI −0.81– −0.19).

In conclusion, maternal glucose values at the pregnancy OGTT were not independently associated with maternal blood pressure outcomes 10–14 years postpartum; however, insulin sensitivity during pregnancy was associated independent of blood pressure, BMI and other covariates measured during pregnancy.

## INTRODUCTION

It is well recognized that gestational diabetes (GDM) is associated with an increased risk of developing type 2 diabetes in the years after delivery (1). More recently, interest has focused on whether GDM predicts the development of other cardiovascular risk factors, including hypertension. A number of studies have investigated whether GDM is associated with the development of hypertension subsequent to pregnancy, but the results of these studies have been conflicting. The majority of studies have limitations such as retrospective reviews using regional, national (2–6) or hospital-based data (7) only, while others obtained data prospectively through maternal self-reporting (8) or through questionnaires administered about 30 years after the original pregnancy (9). Some of these studies also have limitations such as lack of adjustment for potential confounders and a limited length of follow up.

Normal pregnancy is associated with greater insulin resistance and associated metabolic changes. GDM may result in part from an exaggeration of this insulin resistance, but increased insulin resistance may still be present in the absence of GDM. Greater insulin resistance in pregnancy is also associated with hypertension in pregnancy, and this insulin resistance may be present before the onset of hypertension (10). Hypertension in pregnancy is associated with a 2- to 4-fold higher risk of developing hypertension in the long term (11). Metabolic data in pregnancy may provide additional predictors of subsequent hypertension, even in the absence of hypertension during the pregnancy. While insulin resistance may be a useful predictor of subsequent hypertension, we are not aware of studies which have detailed metabolic data in pregnancy enabling study of the relevance of insulin sensitivity to the development of hypertension in the long term. Carpenter (12) noted the need for long-term cohort studies of women who had been well-characterised metabolically in pregnancy. The HAPO Follow-Up Study (HAPO-FUS) provided the opportunity for such follow up of a large cohort of women with detailed examinations during pregnancy and at follow up 10 to 14 years later. The aim of these analyses was to determine whether measures of maternal glucose metabolism are independently associated predictors of subsequent maternal hypertension or whether associations reflect confounders such as maternal BMI and blood pressure during pregnancy.

## MATERIALS and METHODS

The HAPO Study was an observational study designed to examine associations of glucose levels during pregnancy with adverse perinatal and maternal outcomes. HAPO Study methods have been described in detail (13, 14). Briefly, eligible pregnant women underwent a 75-g OGTT between 24–32 weeks’ gestation. Fasting, 1-h, and 2-h plasma glucose (PG), hemoglobin A1c (A1c) and fasting and 1-h C-peptide were measured at a central laboratory (15). All samples were processed at the field center laboratory and shipped to the Central Laboratory. Height and weight were measured using standardized procedures and calibrated equipment. Blood pressure was measured using a calibrated electronic device (Omron 711) on the right arm brachial artery with the appropriate cuff size after sitting quietly for 5 minutes and then a second time after sitting quietly for an additional 1–2 minutes; the mean of the 2 was used for analyses. Women with renal disease were excluded. Demographic and lifestyle characteristics, including age, self-reported race and ethnicity, and smoking and alcohol use during pregnancy, and family history of hypertension and/or diabetes in a first degree relative were collected via questionnaire and parity by medical record abstraction. OGTT results were unblinded for fasting plasma glucose (FPG) > 105 mg/dL (5.8 mmol/L) and/or 2-h PG >200 mg/dL (11.1 mmol/L), or any PG < 45 mg/dL (2.5 mmol/L) (13, 14). A total of 427 (1.8%) HAPO participants had their results unblinded using these criteria and were withdrawn from the study. Blinded participants were untreated. Of the 23,316 participants whose data remained blinded, 2.5% had chronic hypertension, 5.8% gestational hypertension and 4.8% developed pre-eclampsia.

### Participants

For the HAPO FUS, mothers from the HAPO cohort were recruited during 2013–2016 (10–14 years later) from 10 of the 15 original HAPO field centers. Eligibility criteria for HAPO FUS included gestational age at delivery ≥ 37 weeks and no major neonatal malformations or fetal/neonatal death. This yielded 15812 eligible mothers. The recruitment target was 7000 and 4747 participated in the follow-up assessment. Comparison of the characteristics of the participants and non-participants revealed almost identical characteristics for key confounders (16). Of these, exclusions for this study were made for hypertension prior to week 20 of the HAPO pregnancy (n=121), type 1 diabetes (n=4), bariatric surgery (n=49), and cancer treatment (n=1). This left 4572 women for analysis.

The HAPO FUS Protocol was approved by each center’s IRB. All participants gave written informed consent. The study was overseen by an external Observational Study Monitoring Board.

Blood pressure was measured 3 times during the HAPO FUS visit using a calibrated electronic device (Omron 705) on the right arm brachial artery with the appropriate cuff size after sitting for 5 minutes with 1–2 minute intervals between measurements. The mean of the second and third measurements was used for analyses.

### Outcomes and Predictors

The primary outcome for this analysis was a dichotomous variable for maternal hypertension, either identified at follow-up, defined as SBP ≥ 140 mmHg and/or DBP ≥90 mmHg, or by the reported use of antihypertensive medication at the time of the FUS visit. Secondary outcomes were systolic and diastolic blood pressure analysed as continuous variables.

Blood pressure was measured at the time of the HAPO pregnancy OGTT and was analysed as a continuous variable in three ways: mean arterial pressure (MAP), systolic blood pressure (SBP) and diastolic blood pressure (DBP). Blood pressure measurements during pregnancy were used to classify hypertensive disorders in pregnancy according to the International Society for the Study of Hypertension in Pregnancy guidelines (17). Hypertension first diagnosed after 20 weeks’ gestation was treated as a categorical variable comparing categories of gestational hypertension or pre-eclampsia (identified by chart review) to a reference category of no hypertension. Maternal glycemia predictors during pregnancy were GDM, the sum of the glucose z-scores, and insulin sensitivity. GDM was defined by International Association of Diabetes in Pregnancy Study Groups (IADPSG) criteria [one or more glucose values from a 75g OGTT equals or exceeds the following: FPG 92 mg/dL (5.1 mmol/L), 1-h PG 180 mg/dL (10 mmol/L), 2-h PG 153 mg/dL (8.5 mmol/L)] (18). The sum of individual glucose z-scores, an integrated measure that gives equal weight to each of the three glucose values during the OGTT, required calculation of z-scores at each OGTT time point by subtracting the mean glucose level from all HAPO values at that time point, dividing by the SD of the glucose values at that time point, and summing the three individual z-scores. Insulin sensitivity using OGTT glucose and C-peptide (IS_OGTT C-pep_) values was calculated according to the equation of Radaelli et al., with a numerator adjustment for scaling: IS_OGTT C-pep_ = 1,000/ √(FPG × fasting C-peptide ×G ×C), where G and C are the means of fasting and 1-h PG (mmol/L) and C-peptide (ug/L), respectively (19). Continuous predictors were scaled by their standard deviations as estimated in original HAPO data.

### Statistical analyses

Summary statistics from pregnancy were compared for eligible mothers who did and did not participate in the HAPO FUS, including field center summaries weighted by the proportion of the total eligible at each field center (16). Histograms and boxplots were examined to determine the shape of distributions and identify potential outlying observations. Multiple logistic regression was used for dichotomous outcomes; results are reported as odds ratios (ORs) with 95% confidence intervals (CIs). Multiple linear regression was used for continuous outcomes; results are reported as regression coefficients (beta estimates) with 95% CIs.

Models of individual associations of maternal blood pressure were considered first, with maternal MAP, SBP, DBP and hypertension during pregnancy examined for associations with maternal hypertension, SBP and DBP at follow-up, respectively. Next, individual associations were examined for maternal sum of glucose z-scores, GDM and insulin sensitivity with all maternal hypertension and blood pressure outcomes at follow-up. Lastly, joint models of maternal blood pressure and glycemia were examined in pairs as follows: maternal MAP and maternal sum of glucose z-scores, GDM and insulin sensitivity during pregnancy for the maternal hypertension at follow-up outcome; maternal SBP and maternal sum of glucose z-scores, GDM and insulin sensitivity during pregnancy for the maternal SBP at follow-up outcome; and maternal DBP and maternal sum of glucose z-scores, GDM and insulin sensitivity during pregnancy for the maternal DBP at follow-up outcome. Joint models of categorical hypertension predictors during pregnancy with maternal sum of glucose z-scores, GDM and insulin sensitivity during pregnancy were also examined for all follow-up outcomes. All of the analyses described above were performed using a baseline model that included adjustment for known potential confounders and adjustments as already used in HAPO analyses (13): field center (to account for race/ethnicity and other regional characteristics) and maternal variables during pregnancy: age, height, parity, smoking, drinking, family history of diabetes, family history of hypertension, and gestational age and BMI at OGTT. We have previously shown a good correlation (0.92) between pre-pregnancy BMI (based on self-reported pre-pregnancy weight) and BMI at the OGTT (20).

Logistic regression model fit was measured by C-statistics and confirmed by Hosmer-Lemeshow goodness-of-fit tests (21). Linear regression model fit was assessed by scatterplots of residuals vs fitted values, histograms and qqplots of residuals, and DFbeta statistics. Quadratic terms and restricted cubic splines estimated with the *rms* R package (22) were used to assess linearity between the continuous predictor and the log odds of the outcome for logistic regression models and continuous outcomes for linear regression models. Statistical significance was determined according to p<0.05. Analyses presented here are for secondary study outcomes and are not corrected for multiple comparisons. All analyses were conducted in R (3.3.1) (23).

## RESULTS

The characteristics of the 4,572 women during pregnancy and at follow up, stratified by IADPSG GDM status in the HAPO pregnancy, are shown in Table 1.

Table 2 demonstrates that MAP in pregnancy is associated with subsequent hypertension, but that the sum of the glucose z-scores and GDM are not independently associated with hypertension when considered jointly with MAP. In contrast, both MAP and insulin sensitivity are independently associated with hypertension at follow-up when modelled together (OR 2.16, 95% CI 1.90–2.46, p<0.001 for MAP higher by 1 SD; OR 0.79, 95% CI 0.68–0.92, p=0.003 for insulin sensitivity higher by 1 SD).

While maternal SBP in pregnancy is associated with subsequent SBP (Table 3), GDM is not independently associated with SBP at follow-up. Both the sum of glucose z-scores and insulin sensitivity are independently associated with SBP at follow-up. For sum of glucose z-scores higher by 1 unit, SBP at follow-up is higher by 0.22 mmHg (95% CI 0.05–0.38, p=0.01) and for insulin sensitivity higher by 1 SD, SBP is lower by 0.91 mmHg (95% CI −1.34 – −0.49, p<0.001) in models including maternal SBP during pregnancy.

DBP in pregnancy is associated with subsequent DBP at follow-up (Table 3). However, as with the analyses for hypertension, neither GDM nor the sum of glucose z-scores are independently associated with DBP at follow-up when modelled together with maternal DBP during pregnancy. However, insulin sensitivity is independently associated with DBP at follow-up. For insulin sensitivity higher by 1 SD, DBP at follow-up is lower by −0.50 mm Hg (95% CI −0.81 – −0.19, p=0.002) when modelled jointly with maternal DBP during pregnancy.

Both pre-eclampsia and gestational hypertension are associated with hypertension at follow-up (Table 4), but the sum of the glucose z-scores and GDM are not independently associated with subsequent hypertension when considered jointly with the respective hypertensive disorder in pregnancy. In contrast, both hypertensive disorders of pregnancy and insulin sensitivity are independently associated with maternal hypertension at follow-up when modelled together, with OR 2.80 for pre-eclampsia v. no hypertension (95% CI 1.85–4.17, p<0.001), OR 2.60 for gestational hypertension v. no hypertension (95% CI 1.82–3.67, p<0.001) and OR 0.73 (95% CI 0.63–0.85, p<0.001) for insulin sensitivity higher by 1 SD.

Significant departures from linearity for modelled associations in all analyses were not observed.

## DISCUSSION

This study aimed to assess the association between GDM and the subsequent development of maternal hypertension. We have demonstrated that insulin sensitivity in pregnancy is inversely associated with systolic and diastolic blood pressure as well as hypertension 10–14 years postpartum, even after adjustment for multiple confounders from pregnancy including BMI and blood pressure (MAP, SBP and DBP). In contrast, GDM according to IADPSG criteria is not independently associated with subsequent hypertension, SBP or DBP. However, when blood pressure measures are analysed as continuous outcomes, the pregnancy OGTT sum of glucose z-scores was associated with SBP at follow-up independently of SBP in pregnancy; no such relationships were demonstrated for DBP (independent of pregnancy DBP) or hypertension (independent of pregnancy MAP). Although GDM is not independently associated with subsequent SBP and DBP or hypertension, insulin resistance during pregnancy is independently associated with subsequent hypertension as well as SBP and DBP. Similar relationships were demonstrated in women with hypertensive disorders in pregnancy. Multiple measures of blood pressure were evaluated to confirm robustness of findings. In conclusion, identification of women with greater insulin resistance during pregnancy may help to identify those at risk for subsequent hypertension.

Although some studies have shown no association between GDM and subsequent hypertension (4, 7), many previous studies suggested that there may be an association between GDM and subsequent hypertension. However, the data were either collected by questionnaire or retrospectively from a clinical database rather than by direct examination, or the analyses did not take into account relevant potential confounders (2,3,5,6,8,9). We now demonstrate no evidence for an independent association between GDM and subsequent hypertension. The unique features of our study included detailed assessments of the women using standardized protocols both during pregnancy and at follow-up as well as comprehensive metabolic assessments made during pregnancy. Our results suggest that standard measures of glucose during an OGTT may not provide adequate insight into the risk for subsequent hypertension or SBP or DBP levels, but that the degree of underlying insulin resistance may provide more clarity regarding subsequent blood pressure outcomes. Pregnancy is a stress test on a woman’s underlying physiological adaptation and a predictor of subsequent morbidity; thus, we consider our finding that greater insulin resistance in pregnancy, with adjustment for pregnancy blood pressure, is associated with subsequent elevated blood pressure to be a novel and important finding.

While the association of insulin resistance and hypertension outside of pregnancy is well recognised, we are not aware of previous reports on the relationship of insulin resistance in pregnancy and the development of subsequent hypertension. The extent to which the mechanisms underlying insulin resistance in pregnancy differ compared to the non-gravid state is not known. Factors that contribute to pregnancy-induced insulin resistance include the secretion of adipokines (e.g., leptin) and cytokines (e.g., tumor necrosis factor-alpha, interleukin-6, and interleukin-1β), oxidative stress and, possibly, the gut microbiome (24). Recently, we observed similarities in the metabolites associated with insulin resistance in gravid and non-gravid cohorts, although differences in the accumulation of acylcarnitines, which reflects either mitochondrial dysfunction or increased rates of fatty acid oxidation, were observed in pregnancy compared to what has been reported in non-gravid cohorts (25, 26, 27). Thus, despite some apparent differences underlying insulin resistance in pregnant and non-pregnant individuals, greater insulin resistance in pregnancy is associated with subsequent hypertension similar to what has been observed outside of pregnancy. Although the magnitude of the association of greater insulin resistance in pregnancy with blood pressure at follow-up is less than that observed for SBP and DBP during pregnancy, this association may be important for identifying women at risk of hypertension later in life and is of significant scientific interest in potentially furthering our knowledge of the antecedents to hypertension.

While the glucose values during the OGTT identify women at risk of dysglycemia but not hypertension during follow-up (16), greater insulin resistance at the time of the OGTT does identify women at risk of hypertension. With both GDM and hypertension being heterogeneous conditions and as insulin resistance may be present in the absence of GDM, an OGTT in pregnancy combined with C-peptide or insulin measurements could identify those women who are at higher risk of hypertension after delivery due to greater insulin resistance. Just as yearly (28) or 1–3 yearly (29) checks for dysglycemia are recommended for those with abnormal glucose values at a pregnancy OGTT, the simpler measure of annual blood pressure checks could be recommended for those with evidence of greater insulin resistance during pregnancy. However, the practicalities of such a strategy need further evaluation, such as determination of thresholds.

A limitation of this study is that in the original HAPO Study women who gave birth before 37 weeks were excluded. This likely included some who had developed hypertension or pre-eclampsia, but they were not eligible for the HAPO FUS. However, some of these women will have had underlying renal disease and therefore would have been excluded from our analysis. A further limitation is that during the HAPO Study, 1.8% of participants with an OGTT value higher than predefined thresholds were unblinded and excluded from the HAPO Study and thus, the HAPO FUS. These subgroups of women delivered before 37 weeks and those who were unblinded probably included women at higher risk of subsequent blood pressure elevation; accordingly, the reported associations are likely to be underestimates. There are further reasons for considering these as underestimates. Firstly, women with treated hypertension should have had lower SBP and DBP recordings at follow up. Secondly, in the HAPO FUS dropping the first measurement probably provided a fairer representation of “true” blood pressure at follow up, whereas in the HAPO Study the average of the two blood pressure measurements obtained was used, so it is probable that overall “true” blood pressure was marginally lower than the values recorded. Accordingly, the “true” difference in blood pressure between pregnancy and follow-up may be greater than what we have documented. Another limitation is that, without data collection in the intervening period, it was not possible to ascertain whether associations between glucose metabolism during pregnancy and long-term blood pressure outcomes are mediated via other factors. Finally, follow-up of the original HAPO participants over a long interval was a challenge, but the pregnancy characteristics of those who did not attend the HAPO FUS visit were similar to those of HAPO FUS participants.

In conclusion, this study has clarified the controversy in the literature concerning the relationship between GDM and subsequent maternal hypertension. It has shown that although GDM is not independently associated with subsequent elevated blood pressure, underlying insulin resistance is independently associated with subsequent hypertension as well as both systolic and diastolic blood pressure. Furthermore, this study has shown that insulin resistance is associated with these outcomes even in the absence of GDM.

## Figures and Tables

**Table 1:** Characteristics of women during HAPO pregnancy and blood pressure outcomes at HAPO Follow-Up Study according to GDM status

	Overall	GDM	No GDM
	n = 4572	n = 641	n = 3931
Characteristics during Pregnancy	Mean (SD)	Mean (SD)	Mean (SD)
Age (years)	30.0 (5.6)	31.7 (5.3)	29.7 (5.6)
Height (cm)	161.7 (6.8)	160.9 (7.0)	161.8 (6.7)
Weight (kg)	71.4 (13.4)	76.7 (15.0)	70.5 (12.9)
Body Mass Index (BMI) (kg/m^2^)	27.3 (4.7)	29.6 (5.2)	26.9 (4.5)
Mean Arterial Pressure (mm Hg)	80.2 (7.8)	83.0 (7.6)	79.8 (7.7)
Systolic Blood Pressure (mmHg)	104.9 (10.0)	107.9 (10.0)	104.7 (9.9)
Diastolic Blood Pressure (mmHg)	67.8 (8.0)	70.6 (7.8)	67.4 (7.9)
Fasting Plasma Glucose (mg/dl)	81.0 (6.6)	88.9 (7.7)	79.7 (5.4)
1-hr Plasma Glucose (mg/dl)	133.0 (30.1)	172.7 (29.0)	126.5 (24.9)
2-hr Plasma Glucose (mg/dl)	110.3 (23.0)	137.2 (26.7)	106.0 (19.0)
A1c (%)	4.8 (0.4)	5.0 (0.4)	4.8 (0.4)
Fasting C-peptide (ug/l)	1.9 (0.8)	2.6 (1.1)	1.8 (0.7)
1-hr C-peptide (ug/l)	9.6 (3.1)	11.1 (3.4)	9.4 (3.0)
Insulin Sensitivity	3.7 (1.4)	2.5 (0.9)	3.9 (1.4)
Gestational Age (wks)	27.7 (1.7)	27.9 (1.7)	27.7 (1.7)

Race/ Ethnicity	**n (%)**	**n (%)**	**n (%)**
White, Non-Hispanic	2151 (47.0)	255 (39.8)	1896 (48.2)
Black, Non-Hispanic	708 (15.5)	76 (11.9)	632 (16.1)
Hispanic	475 (10.4)	104 (16.2)	371 (9.4)
Asian	1154 (25.2)	190 (29.6)	964 (24.5)
Other	84 (1.8)	16 (2.5)	68 (1.7)
Any Prenatal Smoking	234 (5.1)	39 (6.1)	195 (5.0)
Any Prenatal Alcohol Use	388 (8.5)	52 (8.1)	336 (8.5)
Parity (any prior delivery ≥ 20 wks)	2353 (51.5)	364 (56.8)	1989 (50.6)
Family History of Diabetes Mellitus	1031 (22.6)	195 (30.4)	836 (21.3)
Family History of Hypertension	1737 (38.0)	270 (42.1)	1467 (37.3)
Preeclampsia/Eclampsia	195 (4.3)	55 (8.6)	140 (3.6)
Gestational Hypertension	295 (6.5)	63 (9.8)	232 (5.9)
			
Measures at Follow-Up	Mean (SD)	Mean (SD)	Mean (SD)
Age (years)	41.6 (5.7)	43.4 (5.4)	41.3 (5.7)
Systolic Blood Pressure (mmHg)	113.0 (13.2)	116.2 (12.8)	112.5 (13.2)
Diastolic Blood Pressure (mmHg)	71.7 (9.7)	73.4 (9.1)	71.4 (9.7)

	**n (%)**	**n (%)**	**n (%)**
Systolic Blood Pressure ≥ 140 mmHg	185 (4.1)	34 (5.3)	151 (3.8)
Diastolic Blood Pressure ≥ 90 mmHg	206 (4.5)	33 (5.2)	173 (4.4)
On Antihypertensive Medication	181 (4.0)	46 (7.2)	135 (3.4)
Hypertensive *	410 (9.0)	86 (13.4)	324 (8.3)

* This includes women who were either on hypertensive medication or had hypertension on the basis of their blood pressure measurements

**Table 2:** Associations of mean arterial blood pressure (MAP) and metabolic measures during pregnancy with maternal hypertension at follow-up

	Baseline Models OR (95% CI) p	Adjusted Model: MAP and Glucose Sum of z-Scores OR (95% CI) p	Adjusted Model: MAP and GDM OR (95% CI) p	Adjusted Model: MAP and Insulin Sensitivity OR (95% CI) p
MAP*	2.20 (1.94–2.50) p<0.001	2.19 (1.93–2.49) p<0.001	2.20 (1.94–2.50) p<0.001	2.16 (1.90–2.46) p<0.001
Sum of Glucose z-Scores	1.06 (1.01–1.11) p=0.027	1.02 (0.97–1.07) p=0.45	-	-
GDM (yes v. no)	1.16 (0.87–1.52) p=0.30	-	1.04 (0.78–1.38) p=0.77	-
Insulin Sensitivity^†^	0.71 (0.61–0.83) p<0.001	-	-	0.79 (0.68–0.92) p=0.003

* Odds ratios are presented for the maternal hypertension at follow-up for MAP during pregnancy higher by 1 SD.

† Odds ratios are presented for maternal hypertension at follow-up for insulin sensitivity during pregnancy higher by 1 SD.

All models are adjusted for field center, maternal age, gestational age, height, and BMI at pregnancy OGTT, parity, maternal smoking during pregnancy, maternal drinking during pregnancy, family history of diabetes and family history of hypertension.

**Table 3:** Associations of maternal systolic blood pressure (SBP) and diastolic blood pressure (DBP) and metabolic measures during pregnancy with maternal SBP and DBP at follow-up

	Baseline Models Beta (95% CI) p	Adjusted Model: SBP or DBP and Glucose Sum of z-Scores Beta (95% CI) p	Adjusted Model: SBP or DBP and GDM Beta (95% CI) p	Adjusted Model: SBP or DBP and Insulin Sensitivity Beta (95% CI) p
	**Maternal SBP at follow--up**			
SBP*	4.34 (3.96–4.72) p<0.001	4.29 (3.91–4.67) p<0.001	4.33 (3.95–4.71) p<0.001	4.26 (3.88–4.64) p<0.001
Sum of Glucose z-scores	0.39 (0.22–0.56) p<0.001	0.22 (0.05–0.38) p=0.010	-	-
GDM (yes v. no)	0.94 (−0.12–1.96) p=0.83	-	0.27 (−0.74–1.28) p=0.60	-
Insulin Sensitivity*	−1.36 (−1.81– −0.92) p<0.001	-	-	−0.91 (−1.34– −0.49) p<0.001
	**Maternal DBP at follow-up**			
DBP^+^	3.97 (3.67–4.26) p<0.001	3.96 (3.67–4.26) p<0.001	3.97 (3.68–4.27) p<0.001	3.90 (3.60–4.19) p<0.001
Sum of glucose z-scores	0.18 (0.057–0.31) p=0.005	0.0052 (−0.11–0.12) p=0.93	-	-
GDM (yes v. no)	0.31 (−0.48–1.09) p=0.44	-	−0.29 (−1.02–0.44) p=0.43	-
Insulin Sensitivity^+^	−1.01 (−1.34– −0.68) p<0.001	-	*-*	−0.50 (−0.81– −0.19) p=0.002

* Beta estimates are presented for the difference in SBP at follow-up for SBP and insulin sensitivity during pregnancy higher by 1 SD.

+ Beta estimates are presented for the difference in DBP at follow-up for DBP and insulin sensitivity during pregnancy higher by 1 SD.

All models are adjusted for field center, maternal age, gestational age, height, and BMI at pregnancy OGTT, parity, maternal smoking during pregnancy, maternal drinking during pregnancy, family history of diabetes and family history of hypertension.

**Table 4:** Associations of hypertensive disorders and metabolic measures during pregnancy with maternal hypertension at follow-up

		Baseline Models OR (95% CI) p	Adjusted Model: Hypertension in Pregnancy and Glucose Sum of z-Scores OR (95% CI) p	Adjusted Model: Hypertension in Pregnancy and GDM OR (95% CI) p	Adjusted Model: Hypertension in Pregnancy and Insulin Sensitivity OR (95% CI) p
Hypertension in Pregnancy*	Pre-eclampsia n = 195	3.00 (1.99–4.45) p<0.001	2.93 (1.95–4.35) p<0.001	2.98 (1.98–4.42) p<0.001	2.80 (1.85–4.17) p<0.001
	Gestational hypertension n = 295	2.64 (1.85–3.70) p<0.001	2.60 (1.82–3.66) p<0.001	2.62 (1.84–3.70) p<0.001	2.60 (1.82–3.67) p<0.001
Sum of Glucose z-Scores		1.06 (1.01–1.11) p=0.027	1.05 (1.00–1.10) p=0.07	-	-
GDM (yes v. no)		1.16 (0.87–1.52) p=0.30	-	1.10 (0.83–1.45) p=0.51	-
Insulin Sensitivity^†^		0.71 (0.61–0.83) p<0.001	-	-	0.73 (0.63–0.85) p<0.001

* Odds ratios are presented for maternal hypertension at follow-up for pre-eclampsia/eclampsia and gestational hypertension in pregnancy v. no hypertension during pregnancy.

† Odds ratios are presented for maternal hypertension at follow-up for insulin sensitivity during pregnancy higher by 1 SD.

All models are adjusted for field center, maternal age, gestational age, height, and BMI at pregnancy OGTT, parity, maternal smoking during pregnancy, maternal drinking during pregnancy, family history of diabetes and family history of hypertension

## SUMMARY TABLE

### What is known about the topic

- Whether or not gestational diabetes mellitus (GDM) is associated with subsequent maternal hypertension is controversial.
- There is a relationship both in and out of pregnancy between insulin resistance and hypertension

### What the study adds

- GDM is not independently associated with subsequent elevated maternal blood pressure
- Insulin resistance in pregnancy is independently associated with subsequent maternal hypertension as well as both systolic and diastolic blood pressure
- Insulin resistance is associated with these outcomes even in the absence of GDM.
